# Binaural Sound Localizer for Azimuthal Movement Detection Based on Diffraction

**DOI:** 10.3390/s120810584

**Published:** 2012-08-03

**Authors:** Keonwook Kim, Anthony Choi

**Affiliations:** 1 Division of Electronics & Electrical Engineering, Dongguk University-Seoul, Seoul 100-715, Korea; 2 Department of Electrical & Computer Engineering, Mercer University, 1400 Coleman Avenue, Macon, GA 31207, USA; E-Mail: choi_ta@mercer.edu

**Keywords:** sound localization, rotation detection, diffraction, interaural level difference (ILD)

## Abstract

Sound localization can be realized by utilizing the physics of acoustics in various methods. This paper investigates a novel detection architecture for the azimuthal movement of sound source based on the interaural level difference (ILD) between two receivers. One of the microphones in the system is surrounded by barriers of various heights in order to cast the direction dependent diffraction of the incoming signal. The gradient analysis of the ILD between the structured and unstructured microphone demonstrates the rotation directions as clockwise, counter clockwise, and no rotation of the sound source. Acoustic experiments with different types of sound source over a wide range of target movements show that the average true positive and false positive rates are 67% and 16%, respectively. Spectral analysis demonstrates that the low frequency delivers decreased true and false positive rates and the high frequency presents increases of both rates, overall.

## 1. Introduction

As an acoustic wave travels through a certain medium, the waveform usually contains a variety of cues from the spatial propagation. The proper understanding of this information locates the position of the sound source accurately. In sound localization, the physics of the acoustics are extensive, including signal processing, stochastic processing, *etc.*, by employing the acquired data from amplitude, frequency, and phase of the waveform. Also, the intelligent design of the sender and receiver structure improves the localization performance significantly compared to the isotropic system due to the extra spatial clues.

The conventional sound localization system requires numerous receivers in order to improve the resolution and detection ratio, which is proportional to the number of receivers. However, most animals in Nature perform excellent sound localization tasks with binaural auditory systems which use the comprehensive knowledge of the waveform data. In the human instance, the localization blur is approximately 4 degrees forwards and backwards, and 10 degrees for both sides of the horizontal plane. Moreover, median plane localization performance is acceptable in spite of the high ambiguity of the structure for vertical discrimination [1]. The attained spatial hearing is the result of combining the physical, psychophysical, and psychological aspects of auditory information processing such as interaural level difference (ILD), interaural time difference (ITD), spectral cues, *etc.* [2].

Overall, research on sound localization has been executed in numerous directions, with more than a century of history. The first path is to design a localization system based on the propagation model of acoustics such as the sonar system [3,4]. The second course of exploration is to study the spatial hearing of animals, insects, and fish in order to establish a theory of high resolution algorithms [1,5]. Both of the stated areas are vast and active contemporary subjects of research; however, the third route of sound localization study is fairly novel compared to the others. Connecting both areas and devising the acoustic localizer based on the understanding of the spatial hearing is considered as the third practical area of research. The application of the spatial hearing is distributed over a wide range of technologies including the 3D sound [6], medical diagnosis [7], architectural acoustics [8], and many other applications. The recent novel movement of the research can be described as designing the sound localizer which mimics the spatial hearing of animals with monaural or binaural auditory systems.

The bio-inspired localizer originates from the exhaustive research on spatial hearing and the scope of application includes, but is not limited to, robotics for complementary sensory systems, and the military for surveillance and reconnaissance. In particular, the number of publications related to the topic of intelligent machines with spatial aural perception has gradually increased with the attention to humanoid robots. Rucci *et al.* firstly proposed a computer model of the process of spatial localization of the barn owl to the control of a robotic system [9]. Huang *et al.* described a mobile robot with sound localization for obstacle detection based on the precedence effect model of the human auditory system [10]. In more recent times, the localization is performed by spectral cues given by the artificial head and pinna of the robot body [11–13]. The joint estimation of the binaural sound localization is in progress for enhanced performance of localization based on the time and magnitude of incoming signals [14]. A biological approach based on the spiking neural network model over the ILD and ITD was performed for azimuthal localization by Liu *et al.* and demonstrated the performance improvement from combined information and estimation [15]. High-speed and low power circuitry implementation of an ITD estimator for impulsive signal localization was performed by Chacon-Rodriguez and the paper provides the increased feasibility for complementary sensor systems [16].

In an extension of the stated movement, this paper also contributes to the area of binaural sound localization systems which is potentially applicable to an autonomous robotic system. Basically the authors employ the ILD of a novel structure for detecting the rotation direction of sound source in clockwise (CW) or counter clockwise (CCW) fashion. Unlike the physical structure of the previous papers which duplicate the shape of human or animal hearing systems, this paper fabricates a unique level discriminator named the rotation direction detector (RDD) in the shape of a height changing spiral based on the noise barrier theory [17]. The level difference between the structured receiver and omnidirectional microphone creates the rotational information which is relatively robust to the intrinsic properties of the source spectrum. The simple architecture of the proposed algorithm computes the envelope and ILD of the incoming signal in real time, and the time averaged gradient of the ILD indicates the rotation direction of the sound source. The formation of the RDD is chosen to maximize the ILD and created by a 3D printer for accurate production. The experiments are operated over various sound sources at an anechoic chamber [18], which is in partial conformance with free- and hemi-field performance as defined by ISO 3745 [19].

The objective of RDD system is presented in Section 2, with a focus on the system overview. A design factor and method of RDD system is presented in Section 3. The noise barrier theory, which is the fundamental theory of the RDD structure, is provided to determine physical parameters for the optimal discrimination. The experiments with white noise are analyzed and presented in order to establish the optimal threshold of the detection as well. In Section 4, the performance of the RDD system in terms of the detection ratio is organized. Finally, summary of the strengths/weaknesses and a discussion of future work are presented in Section 5. Note that the RDD and RDD structure indicate the physical structure installed on the microphone and RDD system is the entire system including the microphones, structure, and algorithm.

## 2. Objectives

**Figure 1.**
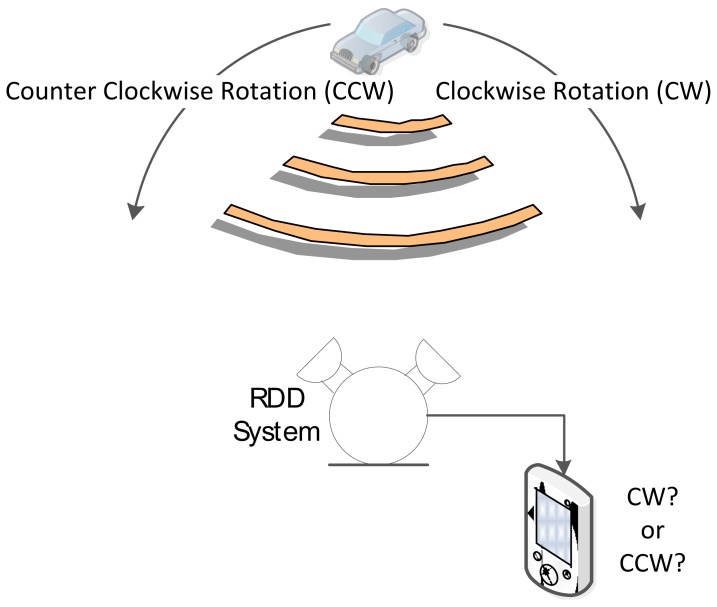
The functional diagram of the RDD system.

The major goal of the RDD system designed in this paper is to identify the direction of azimuthal movement from the sound source by using the two microphones. The relative angular movement of the sound source is specified based on the threshold rather than computing the absolute angular position. The algorithm of the RDD system should be simple and maneuverable for feasibility in terms of the computation and power consumption. Moreover, the profile of the RDD structure is maintained as minimized and low for possible rapid deployment and integration with other systems in the field. The functional diagram of the RDD system is as shown in Figure 1 below.

The conventional multiple-aural methods to localize the sound source are time of flight (ToF) and beamforming. Both of the methods explicitly exploit the temporal information of the incoming signal and isotropic property of the receivers. Since the desired configuration is passive implementation, the ToF method measures the relative difference of the flight time between microphones and computes the angular position based on the simple trigonometry. The higher resolution of time measurement provides the accurate position vector; however, the method is limited to intermittent and broadband signals such as impulse waveforms. The narrow band or continuous sound is supposed to utilize the beamforming method which uses the phase information of the signal. Beamforming is the prevalent technology for localization, but the system requires numerous receivers for proper resolution. In the given implementation, the beamforming algorithm with binaural system barely presents suitable operation due to the wide main lobe of the beam pattern.

For the solution of the given system, the spatial hearing method is applied to recognize the rotation direction of the sound source. The ILD of the binaural receiver is obtained from the level difference between two receivers which have anisotropic and asymmetric characteristic for spatial direction. The intelligent design of the receiver structure denotes the spatial cues which isolate the rotation direction appropriately. The mobilized receiver structure is not considered in this system design; therefore, only the sound source is moved without a priori location information to the receiver. Also, there is no sound dispersion from the RDD system; hence, the detection process is operated silently and passively.

## 3. Methodology

The design of the RDD system includes the three subsections: the fundamental principle, physical structure, and detection algorithm. The primary concept introduced in the RDD system is noise barrier theory which explains the excess attenuation due to the diffraction around a barrier of sound rays emanating from a point source. The fundamental principle is applied to the receiver structure that maximizes sensitivity of the signal attenuation over the direction of arrival (DoA). Finally, the optimal algorithm is presented for ILD and detection in the last subsection.

### 3.1. Noise Barrier Theory

**Figure 2.**
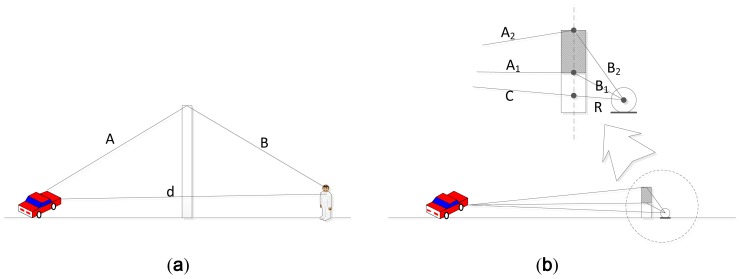
(**a**) Position of source and receiver for conventional noise barrier. (**b**) Position of source and receiver for RDD system.

The level of sound is reduced by an extended barrier as, for example, a wall or a building, and that the obstacle casts an acoustic signal shadow. Nevertheless, the shadow is not ideal because sound is diffracted around the edges of the obstacle. The level attenuation in decibels caused by a height can be calculated for the straight, constant, and infinite length barrier by using the below equation given by Kurze and Anderson [17]:

(1) $$\Delta \text{L}=5\text{dB}+20\text{log}\frac{{\left(2\pi \text{N}\right)}^{\frac{1}{2}}}{\tan\text{h}{\left(2\pi \text{N}\right)}^{\frac{1}{2}}}\text{dB and N}=\frac{2(\text{A}+\text{B}-\text{d})}{\lambda }$$

where (*A*+*B*) is the shortest path length over the edge, from the source to the receiver in the barrier shadow zone and *d* is the direct path distance between source and receiver through the barrier. λ is the wave length of the sound. The parameters are illustrated in Figure 2(a).

The paper [17] describes the sound attenuation by barrier for the wide distance range in order to generalize problem. Note that the distance between the barrier and sound source (or listener) could be near- or far-field for various types of the installation situation. The objective of the RDD system is to provide the rotation-wise information based on the noise barrier theory with the manageable structure profile. Therefore, the receiver is positioned at a nearby barrier and the height of the barrier is modified for individual direction. Also, the sound source is assumed to be remote enough for the far field provision; hence, path between the source and barrier is approximately equal over the height variation. The system and deployment situation is demonstrated in Figure 2(b) in which the distance *A*_1_, *A*_2_, and *C* is roughly alike due to the large distance over the source and barrier. With circular barrier design, the path length of *A*_1_, *A*_2_, *C*, and *R* are approximately invariant for equi-radius moving source in far field condition. The *N* in the Equation (1) is the ratio of the path difference to the wave length and the numerator is modified as below:

(2) $$\text{N}(\theta )=\frac{2({\text{B}}_{\text{n}}-\text{R})}{\lambda }$$

In the equation, *B_n_* is the distance from barrier edge to the receiver and *R* is the length of the direct path from barrier to the receiver. Provided that the RDD is circular shape with height varying structure over the directional angle, the *N* is the function of angle due to *B_n_*. The inclusive level attenuation equation given in Equation (1) creates the output values in terms of incoming angle as well.

### 3.2. RDD Design

**Figure 3.**
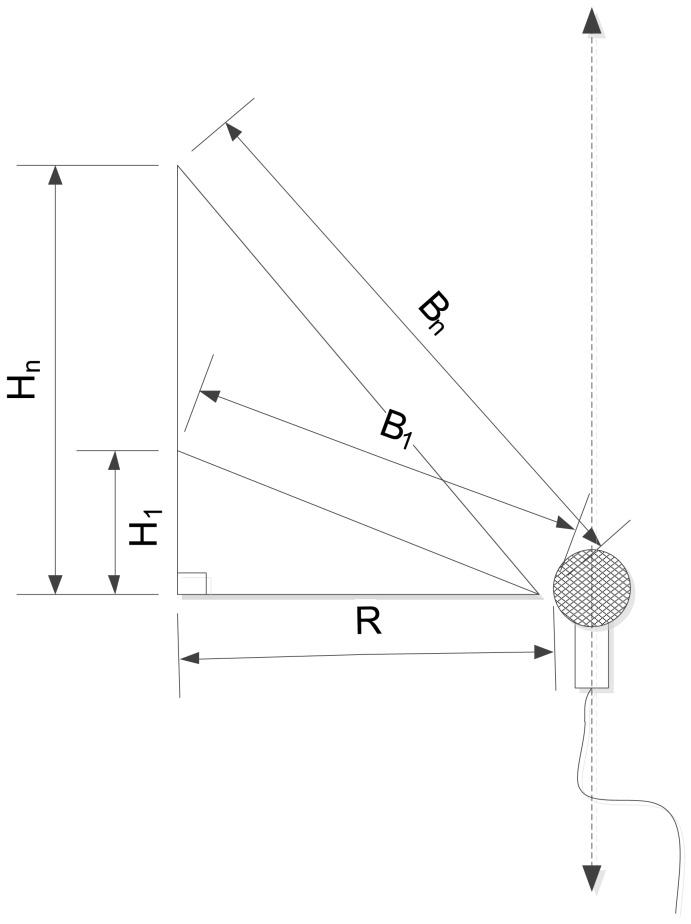
Profile of RDD structure.

The previously described noise barrier problem is transformed into the DoA task by using the height varying barrier. The simple right-angled triangle fulfills the condition described in the preceding subsection as shown in Figure 3. The devised triangle is rotated over the axis parallel to the microphone with constant diameter *R* and variable height *H_n_*. In the perspective of structure pivot, the acoustic signal climbs over the various sizes of hills for individual angle; hence, the experienced path difference produces the distinct level reduction. The height should be chosen for optimal length difference between base and oblique of the triangle in order to generate maximum sensitivity of level reduction in terms of the incoming angle.

**Figure 4.**
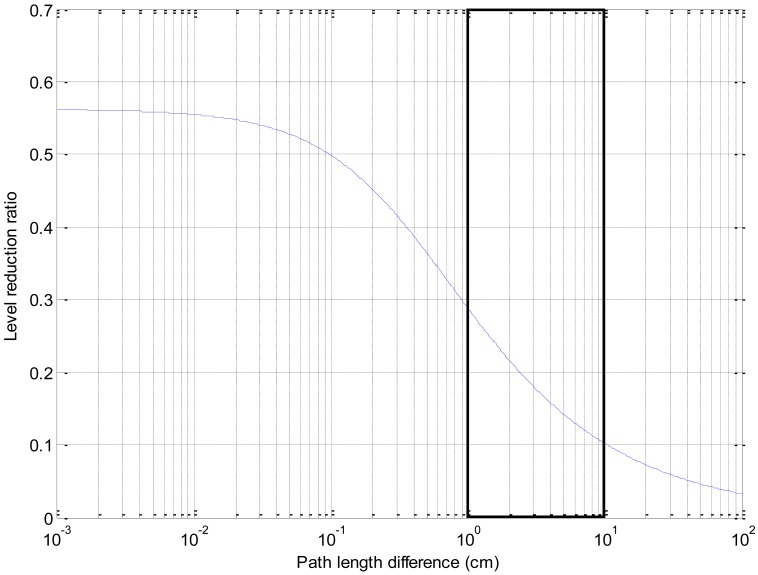
Level reduction ratio of the barrier. Solid box indicates design range of RDD structure.

Using Equation (1), the level of reduction ratio is illustrated in Figure 4 below with input parameter as the path length difference. Notice that the path length difference is proportional to the height length in this configuration because of the fixed receiver location. While the tallness of the barrier is extremely low, the propagated level is relatively low around 55% of the original magnitude due to the diffraction of the acoustic signal over the edge. The practically direct path signal is scattered by the short barrier which establishes the near line of sight between the source and receiver. In Figure 4, the higher gradient indicates the more prominent sensitivity of level variation over the path length difference. Approximately the range from the 3 mm up to the tenths of the centimeter reveals the target scope of the design factor.

**Figure 5.**
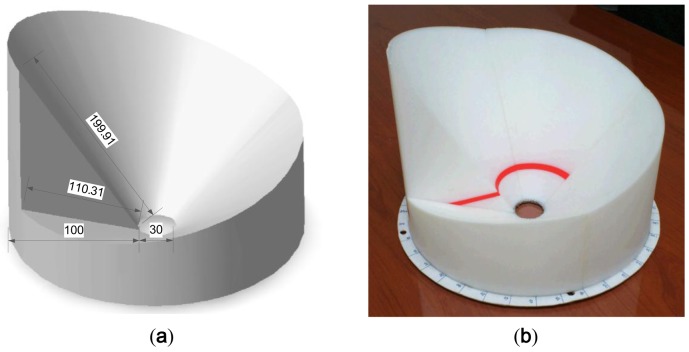
(**a**) Dimension (in millimeter) of RDD. (**b**) Actual structure of RDD.

The design of the RDD is demonstrated in Figure 5 below. The inner hole of the structure is reserved for the receiver location. The profile oblique, which stands for the path between the barrier edge and receiver, is devised to be the extent from 110.31 mm to 199.91 mm along with the outer circumference of the RDD. For the horizontal arrangement of the sound source, the *R* in the Equation (2) is the 100 mm and overall length of the path difference is from the 10.31 mm to 99.91 mm. The simulated level reduction ratio exhibits the value from 0.3 to 0.1 in Figure 4; hence, the graph segment shown in Figure 4 confirms the high gradient area. The final physical structure of the RDD is fabricated by the 3D printer (Dimension; 768 Series) with white ABS plastic material. Note that the entire construction is built by assembling the four small fractions due to the size limitation of the 3D printer.

### 3.3. Detection Algorithm

The monaural configuration of the RDD barely provides the direction information since the receiver cannot divide the level reduction generated whether by intrinsic or extrinsic factor. The co-located reference microphone which receives acoustic signal with isotropic attribute removes the intrinsic level variation by comparison. The binaural design is employed to identify the extrinsic level alteration from the RDD structure properly. The definition of sound level in this paper is defined as the envelope of the signal which can be feasible by the analog or digital method. The envelope signals from the reference and RDD structure are combined and the resulting signal contains the comprehensive DoA information. The gradient of the output indicates the desired rotation direction of the sound source.

**Figure 6.**
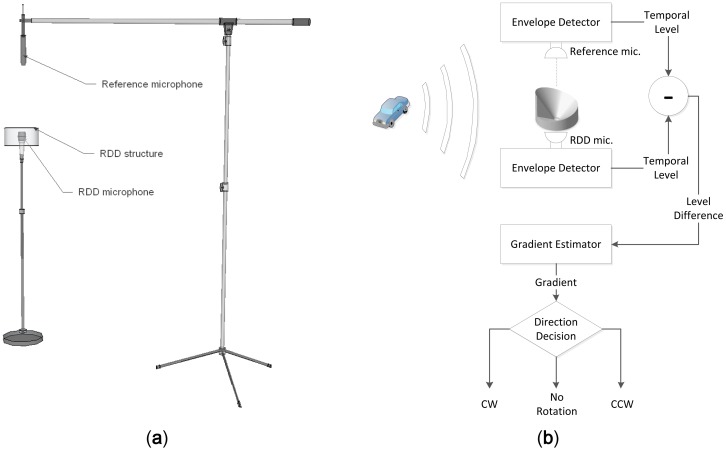
Overall RDD system (**a**) physical configuration and (**b**) detection algorithm.

Figure 6 demonstrates the overall configuration and algorithm for rotation direction detection. The vertical location of the reference microphone shown in Figure 6(a) provides the equi-radius circle to the circular moving target from both microphones. Without the RDD structure, both signals from bottom and top receiver create the identical level for any circular traces. Only the physical structure of RDD imposes the mutual level difference for rotation detection in the system. The envelope detection in the Figure 6(b) is implemented over the digital domain by squaring followed by the first order infinite impulse response (IIR) filter for low pass filtering. The digital low pass filter via first order IIR structure has low complexity but the pole location of the filter significantly approaches to the unit circle for precise filtering. The coefficient should be selected with care for maintaining stability and RDD system utilizes pole location at 0.9999. In order to estimate the gradient accurately, least square method is exercised based on the first order model as *x_n_* = (*a* × *n*) + *b*. The *n* is the discrete time index and *x_n_* is the incoming signal level at the time *n*. The estimated gradient *a* and constant value *b* are obtained by the following equation:

(3) $$\left(\begin{array}{c} \text{a}\\ \text{b} \end{array}\right)={\left({\text{R}}^{\text{T}}\text{R}\right)}^{-1}{\text{R}}^{\text{T}}\text{q},\text{where R}=\left(\begin{array}{cc} 1 & 1\\ 2 & 1\\ 3 & 1\\ \vdots & \vdots \\ \text{N} & 1 \end{array}\right),\text{and q}=\left(\begin{array}{c} {\text{x}}_{1}\\ {\text{x}}_{2}\\ {\text{x}}_{3}\\ \vdots \\ {\text{x}}_{\text{N}} \end{array}\right)$$

The first column of the *R* matrix corresponds to the *n* values up to the data length *N*. Note that superscript *T* denotes matrix transposition and superscript −1 indicates matrix inversion. The uncomplicated version of the gradient estimation such as intermittent sampling can be adapted after the algorithm development stage.

## 4. Analysis

The analysis section provides the interim results for determination of the expecting performance and parameter threshold based on the acoustic experiments. The section is divided into two subsections: structure property and parameter threshold. The structure property presents the experimental result of RDD structure over spatial and spectral domain for verification purpose. The parameter threshold offers optimal limit value determined via the experiments with the white noise and shown by receiver operating characteristic (ROC) curve. Both of the experiments are performed in the anechoic chamber in order to preserve free field condition over the space and frequency field.

### 4.1. Structure Property

The RDD structure is created from the noise barrier theory which investigates the straight, constant, and infinite length barrier with point source. The designed architecture introduces the spiral profile that departs from the assumption described in the theory. Consequently, the verification process is required in order to discover the excess attenuation caused by the RDD structure direction. In this process, the experiment finds the comparative relationship between the height and attenuation rather than the accuracy of the Equation (1). Spectral and spatial exploration is executed and analyzed in the anechoic chamber which has been verified to exhibit partial conformance with ISO 3745 for free field and hemi-free field condition [18].

**Figure 7.**
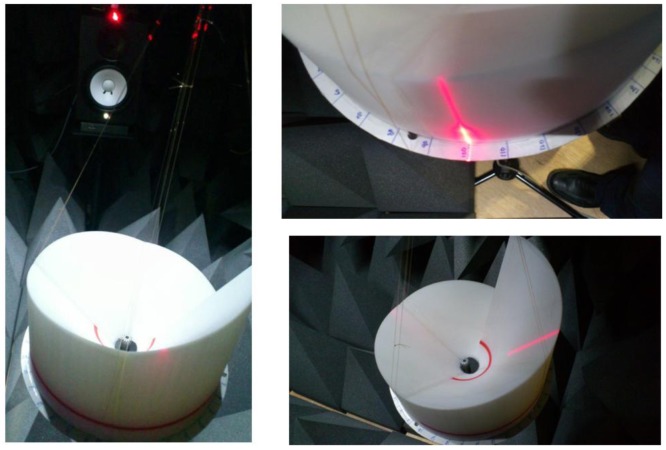
Acoustic experiment in the anechoic chamber with RDD structure.

The equipment and configuration used in the experiments are listed as follows. The speaker (Yamaha; HS80M) is an active studio monitor speaker with an 8″ low frequency driver and a 1″ high frequency driver. The speaker can generate up to 120 watt power over the range from 42 Hz to 20 kHz and is connected by the balanced XLR cable. The microphone (Behringer; ECM8000) is a condenser microphone for measurement with flat frequency response and omnidirectional pattern. The microphone works with phantom power and is connected by balanced XLR cable. The audio device (Cakewalk; Sonar V-Studio 100) is a multiple input and output audio device which can handle up to 8 inputs/6 outputs with 24 bits/96 kHz sampling quality. The device is connected by USB and driven via ASIO 2.0 driver. The computer software for mixing and capturing is Sonar VS from Cakewalk. The laser level meter (Black & Decker; BDL220S) located on top of the speaker radiates the vertical laser surface for indicating the DoA angle which is estimated by the protractor placed in the bottom of the RDD structure. The distance between the microphone end and the speaker driver is 1.2 m. As shown in Figure 7, the RDD structure is suspended over the microphone with fishing wire from the chamber ceiling; therefore, the RDD structure rotates freely in the acoustically transparent condition.

**Figure 8.**
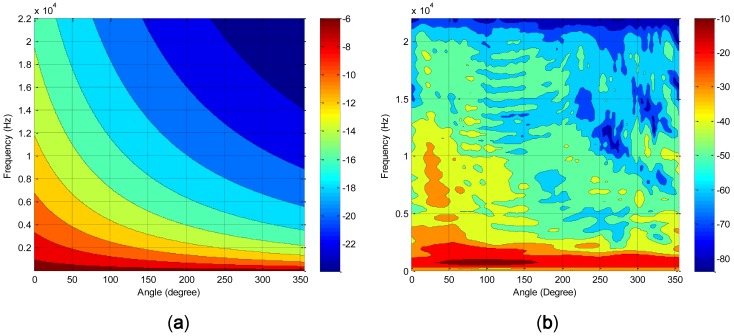
(**a**) Theoretical spectral distribution of RDD structure. (**b**) Experimental spectral distribution of RDD structure.

The acoustically whitened signal based on the chamber transfer function is transmitted and recorded in five degree increments in order to generate the spectral distribution. From Equation (1), the theoretical spectral distribution for 360 degrees is illustrated in Figure 8(a), which denotes the significant level reduction at the elevated barrier height. It should be noted that the height of the barrier is proportional to the angle of the structure. In the vicinity of high angle, the spectrum is covered by low level values in particular for high frequency range. Also, the high level values become narrower for the low frequency range. The experimentally measured spectral distribution is exhibited in Figure 8(b) with 128 sample window length and ensemble averaged in order to reduce the variance of the signal. The spline interpolation with five times grid expansion in angle and frequency is used for Figure 8 in order to draw the smooth contours representing the comprehensive characteristics of the spectrum field. Unlike the theoretical result, the evaluated outcome represents scattered distribution of the contour plot with significant loss of energy at the high frequency. Due to the frequency limitation of the speaker, the high pitch component is suppressed and reduced in the graph over the 20 kHz. The overall distribution of the sound level approximately corresponds to the theoretical graph since the color allocation can be described by the diagonal line, which is illustrated in the counterpart image as well. Moreover, the additional energy loss can be observed for the further movement toward the higher angle. Both of the figures confirm the relationship between barrier height and sound level by means of acoustic experiment in the anechoic chamber.

**Figure 9.**
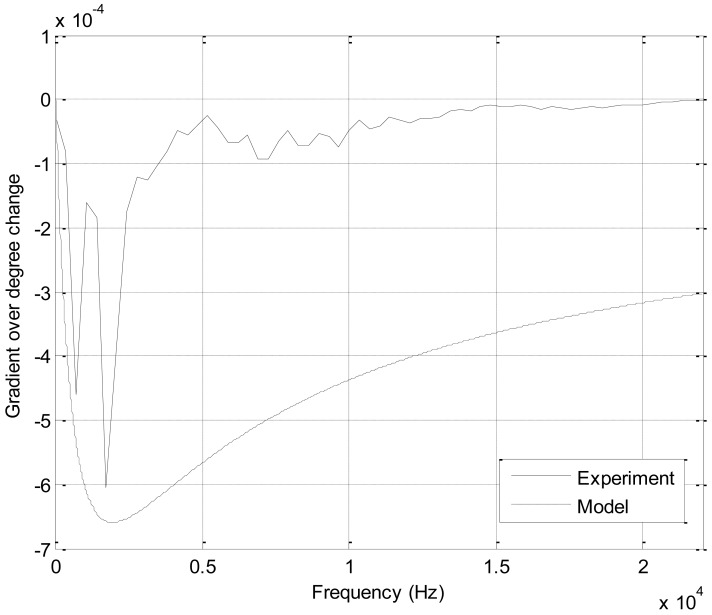
Gradient of the level distribution over the entire angle.

The level reduction ratio can be examined by computing the horizontal slope of Figure 8, which shows the sensitivity of the sound level for individual frequency in terms of angle. The increased sensitivity provides the improved the detection ratio due to the higher level variation from the given angle deviation. Figure 9 demonstrates the gradient of the level distribution over the entire angle for individual frequency. In Figure 9, the slope has negative values due to the inversely proportional relationship between the sound level and angle (or height). The slope of measured and model show the higher absolute values around 2 kHz and rapidly declining trails toward high and low frequency. At the higher frequency, the significant loss of the intensity from measurement establishes the visible departure between the experiment and model plot. The mid range frequency around several kilo hertz contains the essential clues in order to discriminate the directional movement of the sound source based on the RDD structure. The breadth of the frequency used for the detection is determined by the further experiment from the subsequent subsection.

### 4.2. Parametric Threshold

The designed algorithm of the RDD system requires determining the values of two parameters which are frequency range and time interval for rotation direction detection. The frequency range is the spectral scope manipulated for entire process and the time interval is the temporal length speculated for gradient estimation. The optimal values of the variables are estimated by the statistical process based on the experiment that considers wide range of target movement. The outcome of the probability analysis provides the favorable values representing the high detection and low false alarm rate for the given experimental set. Consequently, the subsection denotes the procedure of selecting the threshold values for the spectral and temporal parameter.

The movement of sound source can be described by the polar coordinate system in which each source position on a plane is determined by a radius and azimuth with the RDD microphone located at origin. Since the Euclidean distance is described by the inverse square law in the acoustic propagation, the radius variation is expressed by signal level which depends on the position and velocity. The diverse velocity and position in radius direction is realized by employing the derived envelope to the given signal. The azimuthal movement is achieved by rotating the RDD structure in order to change the signal DoA instead of repositioning the source location. With stationary acoustic sender, the horizontal movement of the sound source is accomplished by signal envelope and RDD structure rotation in the medium profile anechoic chamber.

**Figure 10.**
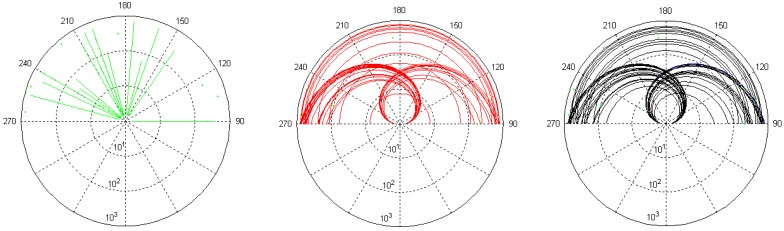
Entire trace of sound source in polar coordination with degree angle and meter radius. Green → no rotation, red → CW, and black → CCW.

Based on the uniform distribution random variable, the experimental data set is generated for the wide range of rotation, velocity, and position with equal likelihood. Figure 10 presents the 100 traces of sound source for the given experiment in polar coordination system and the red, black, and green lines reveal the rotation direction for CW, CCW, and no rotation respectively. The straight and spiral lines in Figure 10 illustrate the sound source with radial velocity, which is constant for the given time span. The sound source dissipates the signal for 30 seconds and the maximum radial velocity is 97.08 km/h in this experimental configuration. The rotation angle is fixed as the range from 90° to 270° due to the limitation of structure rotator which provides the DoA variation in a continuous and silent manner. The hanging RDD structure is connected by fishing wire at the position of 90° and 270°, and the wires are routed to the exterior of the chamber through a plastic sleeves on the wall. By pulling one of the wires, the RDD structure is rotated in CW or CCW direction. Due to the human operator, the angular velocity of the RDD structure is likely to be irregular; however, the structure is rotated with near silence.

**Figure 11.**
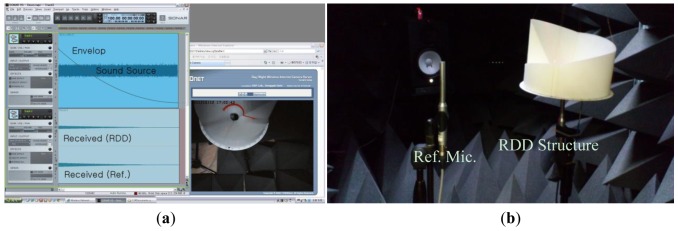
(**a**) Computer screenshot. (**b**) Acoustic experiment in anechoic chamber.

In the RDD experiments, the two microphones are arranged to be parallel in the anechoic chamber and one of the receivers is installed with the RDD structure as shown in Figure 11(b). Since the azimuthal movement of the target is derived from the RDD structure rotation only, the horizontal configuration of the reference microphone is also provide the identical situation as the vertical arrangement shown Figure 6(a). The speaker emits the whitened noise signal with envelope, which simulates the time varying radial position of the sound source. The computer software (Cakewalk; Sonar VS) and the audio device (Cakewalk; Sonar V-Studio 100) have the capability to control the level of output signal by placing the track envelope that manipulates the motorized volume fader. The envelope graph is derived from the inverse square law with logarithm function and applied to the specific output track. While the azimuthal movement of the RDD structure is executed by the human operator, visual supervision is performed for improved angular control by using the wireless video camera. Figure 11(a) shows the computer screenshot, which visualizes the audio software and top-view RDD structure. The real-time visual feedback of the DoA angle fulfills the coarse manipulation of angular velocity for the given experimental interval.

**Figure 12.**
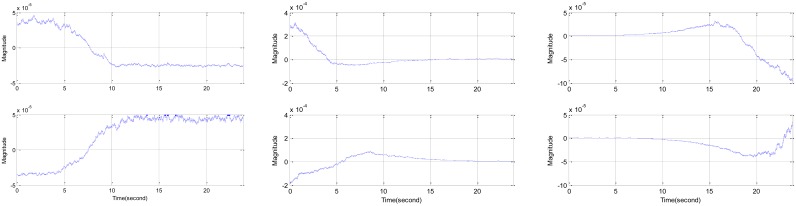
Disparity level between the RDD and reference signal for constant distance (**left**), withdrawing (**middle**), and approaching (**right**) target. The above and below figure are for CW and CCW direction respectively.

According to the experimental configuration described above, the disparity levels between the RDD and reference signal are generated for the selected movement situations in Figure 12. It should be noted that the signal is processed with the envelope detector; therefore, the high frequency component is reduced for corresponding to the approximate signal level. The rotation of CW direction increases the DoA angle, which relates to the higher barrier height; hence, the disparity level tends to have a negative gradient as shown in the first row of figures. The CCW direction is demonstrated at the second row figures with positive gradient due to the lower barrier height for the further rotation. All depicted figures represent the correct rotation direction given by the acoustic signal in the distinctive radial movement. The left column delivers the situation of the equi-radius angular movement. The middle and right column provide the condition of the radial movement for the withdrawing and approaching source respectively. The overshoot and undershoot in the figures are caused by the signal level transition and IIR filter since the filter in the envelope detector has extended convergence time for the input signal change. Note that no significant shoots are observed at the left column situation because of the steady input level from equi-distance movement.

**Figure 13.**
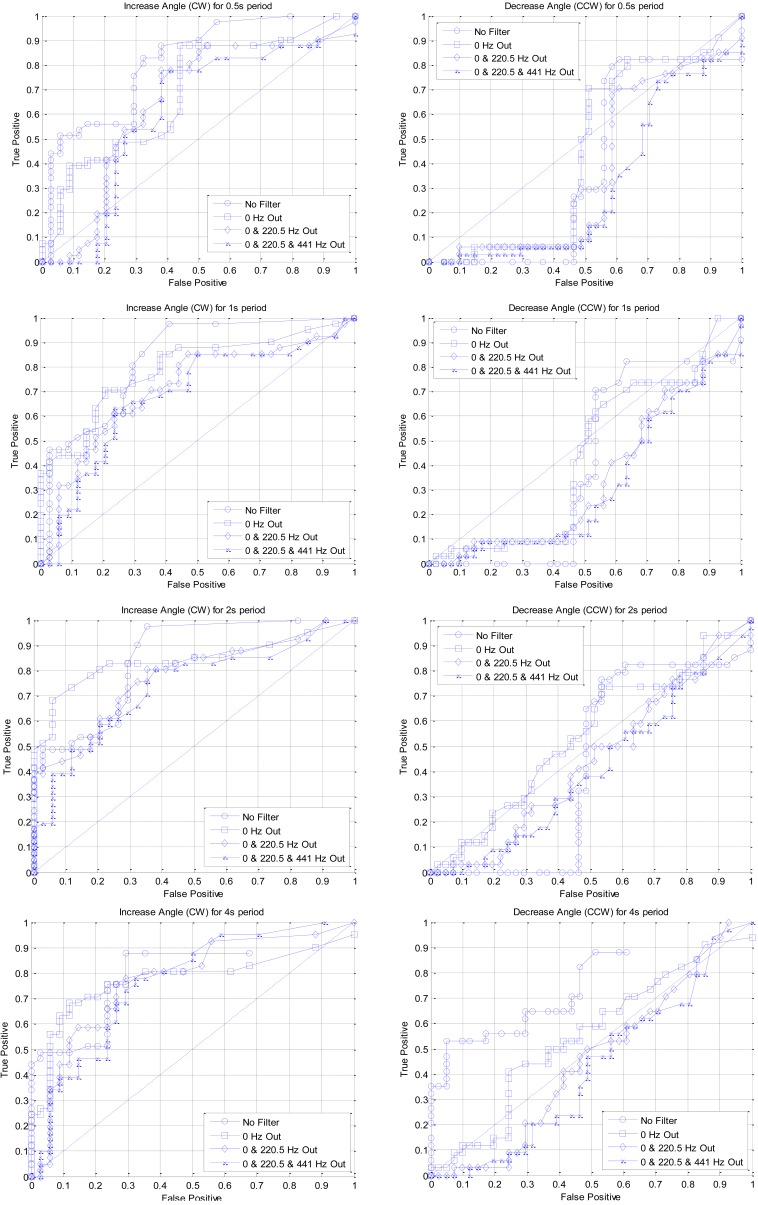
ROC curve for individual rotation direction, time window, and processing frequency.

The ROC curve for the given experimental set is expressed in Figure 13. The column and row of the figure in the cluster stand for rotation direction and time window length respectively. Each plot within the figure is generated from the distinctive range of the processing frequency which is whole or selectively cut frequency. The span of the time window is from a half second to four seconds without overlaping for estimating the output gradient that provides the decision criterion. The filter configuration for frequency selection is designed based on the finite impulse response (FIR) filter by placing the zeros onto the specific location on the unit circle. The order of the FIR filter is determined by the number of zeros which is associated with suppressing frequency; therefore, the number of the removed frequency is proportional to the filter order in the given configuration. Accordingly, the devised filter is the high pass filter, which removes the low frequencies around the several hundred hertz. Note that further selection of eliminated frequency indicates the increased range of removal for low frequency.

According to the ROC performance, the curve approaching to the upper left corner signifies the improved detection operation. In the ROC results, visible performance bias is observed between the CW and CCW rotation due to the coarse manipulation of physical operation. Consequently, the mean value of the CW angular velocity is higher than the expectation value of CCW rotation speed by pulling the one of the wires from the chamber outside. Further analysis of the rotation speed indicates that asymmetric position of pulling string creates the inclined rotation speed for each direction. The manual control for consistent rotation speed is impractical even with careful consideration of the pulling mechanism and visible feedback. Including the variation created by human operation, the bias from the structural imbalance is regarded as the diversity of experimental set in order to derive the statistically informative outcome. Note that the randomly generated data set specifies the radical speed and rotation direction only; therefore, the angular velocity is arbitrarily imposed by human operator.

The column-wise analysis of Figure 13 represents the detection rate investigation in terms of the time interval as a half, one, two, and four seconds. The increased range of the temporal window provides the taller plot to the upper left corner that indicates the advanced statistical performance. Both of the rotation direction shows the performance improvement with extended time window and the CCW direction offers the further relative enhancement than baseline result. Especially, the true positive rate in the reduced threshold is significantly improved for the wider window length as shown in the lower left corner of the ROCs in CCW direction. Further increase of the time window expects to generate the enhanced RDD performance with the given testbed data since the data group consisted of the monotonically rotating objects.

As shown in Figure 9, the low frequencies contain the essential discriminating information for the angular direction of the RDD system. For verification purpose, the range of the low frequencies is eliminated from the processing subsequent to the envelope detection stage and statistical performance is measured by ROC curves in the figures. The performance of the individual signal seems to be random in the certain part of the ROC graph; however, the signals with reduced removal generally show the superior functioning, which is revealed by the position nearest to the upper left corner. By inspection of the given figures, the four second time interval with no high pass filter is chosen for the gradient estimation. The precise threshold values for the gradient are computed by the shortest Euclidean distance between the ROC curve (four seconds and no filter) and the upper left corner. The ROC for the CW and CCW direction provides negative and positive threshold values respectively. Higher than the positive threshold indicates the object is rotated in the CCW direction and lower than the negative threshold specifies that the source is rotated in the CW direction. The value between the positive and negative threshold means no rotation including the pure radial movement.

## 5. Experiment Results

**Figure 14.**
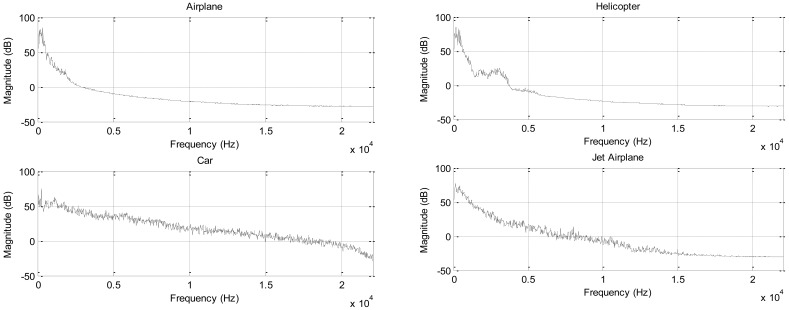
Spectrum of sound sources.

Previous analysis is performed based on the white noise signal, which is fabricated by the normally distributed random numbers, for structure verification and parameter estimation. To evaluate the RDD system, the realistic sound data representing the conventional situation is required for statistical analysis. The general sound from outdoor field is most likely to change the spectrum over time; therefore, the stationary spectrum is devised to execute the experiments in order to derive statistically consistent outcome. Four sound sources selected are airplane, helicopter, car, and jet airplane which are recorded at the fixed location over the moving object. Range of time data that corresponds to the clear representation of acoustic characteristic is used as the FIR filter coefficient for generating the stationary sound source based on the white input signal. The spectrum of the given data set is illustrated in Figure 14. The frequency distribution of the airplane and helicopter demonstrates the relatively narrow band signal concentrated on the low frequency region. On the other hand, car and jet airplane spectrum displays extensive distribution of the power over the wide frequency range. Therefore, the individual sound source denotes the distinctive spectrum sound for improving the diversity of the experiment. Also, note that the volume of each signal is unified at the time of wave creation.

The configuration and deployment of the acoustic experiment identically follows the execution specified in Subsection 4.2, except the sound source. The source movement is described by Figure 10 for each acoustic source and the RDD system produces the output of rotation direction based on the threshold derived from the previous section. Table 1 organizes the result of the detection rate for individual sound source. Overall the average true positive and false positive rates are 67% and 16%, respectively. Due to the manual operation for the angular movement, CW direction has approximately 15 percentage point (pp) further true positive rate and 6 pp less false positive rate on average. Moreover, the table presents the tendency of proportional relationship between the true and false positive rate. The characteristic of the incoming signal creates the distinctive probability distribution with considerable overlap between the distributions; hence, the division from the optimal threshold expects to show the high false positive rate.

The individual signal shows the unique performance attributes which depend on the spectral feature of the signal. Low frequency oriented signal, such as the airplane and helicopter, demonstrates the low true and false positive rate as 57% and 1% correspondingly. Comparatively, the flat spectral signal for the car and jet plane establishes high true and false positive rate as 77% and 32% in that order generally. Therefore, a low frequency component contributes to decrease the false positive rate with sacrificing the true positive rate and the high pitch element lends itself to increase the true positive rate with surrendering the false positive rate. The airplane sound provides the spectral signal mainly focused on the low frequency and the signal creates the around the 56% true positive rate and 0% false positive rate. On the other hand, car sound presents the relatively even spectrum over the wide range and the signal delivers approximately 75% true positive rate and 20% false positive rate.

The optimal threshold values selected from the ROC curves are developed over the extensive range of the frequency; hence, the beneficial detection rate presents high true and false positive rate as the case of the car sound. According to Table 1, the stated relationship between the spectral input and statistical output is convinced and extended to the distinct properties of acoustic signal. The featured sound with low frequency expects to generate improved performance as a result of the high gradient over the barrier height change shown in Figure 9. The high frequency component significantly contributes to increased false positive rate as shown in the case of the car and jet airplane. Therefore, the designated low pass filter prior to the gradient estimation anticipate to progress the detection performance with high true and low false positive rate, which is near to the upper left corner in ROC curve. Additional analysis is required to achieve the detection outcome based on the particular frequency selection in order to improve the performance of the RDD system further. The spectral manipulation for the gradient estimation via the application of dedicated filter is left to the future research for the elaborated rotation detection.

## 6. Conclusions

This paper presents the design and analysis of a novel rotation direction detection system based on the binaural architecture. The noise barrier theory is adopted to design the discriminating scheme between two microphones; hence, spiral physical structure applied to one of the microphone produces the direction dependent output. Upon the DoA, the path difference between the direct and indirect propagation establishes the spectrally reliant level reduction. The level difference between the reference and structured microphone provides the criterion of the rotation direction. Instead of using the instantaneous value of the difference, the estimated gradient is employed to determine the rotation direction for reducing the false detection rate due to the signal glitch. According to the designed configuration, the system only senses the azimuthal rotation as CW, CCW, and no rotation.

The decision criterion is provided by the gradient of the level difference between two distinct receivers. The optimal parameters for the gradient estimation are statistically decided by using the experiments with white noise signal in the anechoic chamber. By the inspection of the ROC curves, the four second time interval with no high pass filter is chosen for the gradient estimation. The acoustic experiments with different type of sound source over the wide range of target movement show that the average true positive and false positive rates are 67% and 16%, respectively. The low frequency oriented signal demonstrates the low true and false positive rate as 57% and 1% correspondingly. Comparatively flat spectral signal establishes high true and false positive rate as 77% and 32% in that order. Spectral analysis demonstrates that low frequency delivers decreased true and false positive rates and the high frequency increases both positive rates, overall.

The novel detection architecture for azimuthal rotation illustrates the desired statistical performance for various targets in horizontal movement. The detection system described in this paper presents simple and maneuverable solution for localizing the target rotation direction over the conventional multiple-aural system, which requires the complicated digital architecture in order to extract phase or time information. The loosely coupled binaural receivers from the envelope detector expect to conserve operation energy due to the low sampling rate given by the smooth output signal; however, this is not analyzed here. Future work will involve performing the researches on the design of the dedicated filter prior to the gradient estimation for improving detection performance further. Moreover, the granted mobility of the structure will promote the reduced complexity architecture as the monaural form and the mobile localizer with advanced algorithm anticipates locating the rotation as well as the direction of the sound source accurately.

## Figures and Tables

**Table 1.** Detection performance for various sound sources.

**Sound source**	**Rotation direction**	**True positive rate (%)**	**False positive rate (%)**
Airplane	Dec. angle (CCW)	52.94	0.00
	Inc. angle (CW)	58.54	0.00
Car	Dec. angle (CCW)	64.71	34.15
	Inc. angle (CW)	85.37	5.88
Helicopter	Dec. angle (CCW)	47.06	0.00
	Inc. angle (CW)	68.29	2.94
Jet airplane	Dec. angle (CCW)	73.53	43.90
	Inc. angle (CW)	85.37	44.12
